# Uncovering and investigating reasons for inaccuracies in vaccine card records in Kiyawa LGA Jigawa, Nigeria: a mixed method study

**DOI:** 10.1186/s12913-026-14826-2

**Published:** 2026-06-06

**Authors:** Ayodamola Bakare, Julius Salako, Funmilayo Shittu, Ayobami A. Bakare, Obioma Uchendu, Hamish R. Graham, Eric D. McCollum, Agnese Iuliano, Rochelle A. Burgess, Samy Ahmar, Tahlil Ahmed, Adamu Isah, Magama Abdullahi, Osebi Adams, James Beard, Adegoke G. Falade, Carina King, Tim Colbourn

**Affiliations:** 1 Department of Paediatrics, University of Ibadan, Ibadan, Oyo State Nigeria; 2 Institute for Global Health, University College London, London, UK; 3 Department of Global Public Health, Karolinska Institutet, Stockholm, Sweden; 4 Department of Community Medicine, University College Hospital, Ibadan, Oyo State Nigeria; 5 Department of Community Medicine, University of Ibadan, Ibadan, Nigeria; 6 Centre for International Child Health, Murdoch Children’s Research Institute, University of Melbourne, Royal Children’s Hospital, Parkville, VIC Australia; 7 Eudowood Division of Pediatric Respiratory Sciences, Department of Pediatrics, School of Medicine, Johns Hopkins University, Baltimore, USA; 8 Save the Children UK, London, UK; 9 Save the Children Nigeria, Abuja, Nigeria; 10 Independent Consultant, Guildford, UK; 11 Department of Paediatrics, University College Hospital, Ibadan, Oyo State Nigeria

**Keywords:** Vaccination card accuracy, Healthcare worker, Immunization records, Caregiver’s recall, Low- and middle-income countries

## Abstract

**Background:**

Accurately measuring vaccination coverage is crucial for programmatic and policy decision making, however accurate measurement of coverage can be challenging. We aimed to understand the extent of, and reasons for, inaccurate vaccination card records in a rural, low-income setting in Jigawa state, Nigeria.

**Methods:**

We conducted an explanatory sequential mixed-methods study in Kiyawa Local Government Area, Jigawa State, from September 2022 to July 2023, using data from the INSPIRING Jigawa trial (ISRCTN39213655). Quantitative data was gathered from surveyed women aged 16–49 in sampled compounds, who presented their child’s vaccine card. Vaccine documentation was defined as inaccurate when the vaccine card is discordant with caregiver report. Adjusted logistic regression identified factors associated with inaccuracies. We conducted in-depth interviews with healthcare workers and focus groups with community members, analyzing transcripts using conventional content analysis, and triangulated findings with quantitative results.

**Results:**

Records for 4258 children under five-years, from 3232 women, were examined. Of these children, 441 (10.4%) had vaccine cards that were deemed inaccurate by their caregivers. Inaccuracies were primarily attributed to cards being filled despite the child not receiving the vaccine, misplacement or loss of vaccine cards, vaccine stock-out when cards had already been filled, and vaccine card stock-out when the vaccine had been administered. Our adjusted logistic regression results show the following variables were associated with reporting inaccurate vaccine cards (under or over-reporting): any education compared to none (adjusted odds ratio (aOR): 1.33, 95%CI: 1.03, 1.75), having co-wives compared to no co-wife (aOR: 0.78, 95%CI: 0.62, 0.98), and child’s age: 12–24 months compared to < 12 months (aOR: 2.70, 95%CI: 1.94, 3.75) and 25 months and above compared to < 12 months (aOR: 2.30, 95%CI: 1.69, 3.12). Our qualitative findings highlighted maternal lack of knowledge of vaccination schedule and forgetfulness about the vaccination schedule as common reasons for vaccine card inaccuracy.

**Conclusion:**

We identified health system failures and caregiver barriers leading to inaccurate reports in vaccine cards. It is essential to sensitize caregivers and healthcare providers on the importance of accurately documenting vaccines and validating immunization recording systems.

**Clinical trial number:**

Not applicable.

**Supplementary Information:**

The online version contains supplementary material available at 10.1186/s12913-026-14826-2.

## Background

Globally, immunization programs have made significant strides in reducing the burden of infectious diseases such as tuberculosis, polio, pneumonia, diphtheria, pertussis, tetanus, hepatitis B, and measles. However, persistent disparities in vaccine access and delivery, particularly in low- and middle-income countries (LMICs), continue to hinder progress. In 2023 an estimated 14.5 million infants failed to receive the first dose of the diphtheria-tetanus-pertussis (DTP) vaccine, with an additional 6.5 million children remaining only partially vaccinated. This gap in immunization coverage contributes to the 4.9 million under-five child deaths due to preventable causes each year [1, 2]. These gaps in coverage are most pronounced in sub-Saharan Africa, where weak health systems, socioeconomic inequities, cultural and logistical barriers exacerbate low vaccination coverage rates [3, 4].

Accurate and reliable vaccination coverage data is essential for addressing immunization gaps. Coverage estimates are generally derived from two primary sources: Routine administrative data, and Household surveys using immunization cards and caregiver’s recall, each presenting unique strengths and limitations. Household surveys, such as the Demographic and Health Surveys (DHS), provide robust population-level estimates and are considered a gold standard, but their resource intensity and infrequent implementation limit their real-time utility [5, 6]. Routine administrative data offer timely updates, but are prone to inaccuracies due to reporting inconsistencies, population estimation errors, and potential data manipulation, impacting their reliability for program management [7–10]. Immunization cards serve as vital tools for tracking individual vaccination histories, enabling informed clinical decision-making, preventing unnecessary vaccinations, and promoting caregiver adherence. Although immunization card data are essential for public health surveillance, pinpointing areas with low coverage, and directing targeted interventions, caregiver recall can serve as a necessary alternative for estimating childhood vaccination status when vaccination cards are unavailable or deemed unreliable. Studies suggest that caregiver recall can be a dependable method, although its accuracy varies depending on the context and type of vaccine [11, 12].

Nigeria is estimated to have the highest number of ‘zero dose’ children, and the highest number of absolute child deaths each year, linked to systemic weaknesses in Nigeria’s health infrastructure, shortages in the healthcare workforce, and logistical barriers in vaccine distribution [13–15]. As a result, vaccination coverage remains critically low. The 2024 Nigeria Demographic and Health Survey (NDHS) reported that only 39% of under-5 children nationwide, and 56.9% in Jigawa State, received at least one dose of BCG vaccine for tuberculosis protection, three doses of polio vaccine, three doses of DPT-containing vaccine against diphtheria, pertussis, and tetanus, and one dose of measles-containing vaccine. The survey assessed vaccination coverage for these basic antigens by calculating the percentage of children who received specific vaccines at any time prior to the survey, as documented by either vaccination cards or maternal report. These figures indicate Nigeria is off track for achieving Sustainable Development Goal (SDG) 3.8 target of achieving universal health coverage for vaccination by 2030 [16, 17].

Jigawa State, in Northern Nigeria, faces distinct contextual challenges that severely compromise the reliability of immunization card data [18, 19]. The increased drive to improve vaccine coverage has led to reports of some unintended consequences. Financial incentives leading to increased demand has overburdened healthcare workers, leading to inadequate record-keeping and inaccurate card completion [20, 21]. Moreover, these incentives foster an environment where caregivers may coerce healthcare workers into falsifying vaccination records, resulting in distorted coverage estimates and a critical loss of data integrity [19, 22]. Given the absence of electronic registries, vaccination cards are the exclusive tool for vaccination coverage estimation in Jigawa. Reliance on vaccination cards risks perpetuating inaccurate vaccination coverage estimates and misguiding resource allocation. Therefore, the objectives of this study are to: (i) quantify discrepancies between vaccine coverage estimates from vaccination cards and maternal recall; and (ii) investigate systemic and contextual factors that contribute to vaccine card inaccuracy, for children under 5 in Kiyawa LGA, Jigawa State. By identifying these factors, and the scale of vaccine card inaccuracy, this research will generate evidence-based recommendations to strengthen immunization data quality, and support accurate monitoring of vaccination uptake and global health targets.

## Methods

### Study design

This study employed an explanatory sequential mixed-methods design. Initially, quantitative cross-sectional data was gathered from women aged 16–49 with children under five in the Kiyawa Local Government Area (LGA) of Jigawa State, Nigeria, between September 12, 2022, and January 25, 2023. This data was collected as part of the endline survey for the INSPIRING Jigawa cluster randomized controlled trial (ISRCTN39213655) [23]. We then collected qualitative data to explore factors contributing to vaccine card inaccuracies through Focus Group discussions (FGDs) with male and female caregiver groups in selected Kiyawa LGA communities, and in-depth interviews (IDIs) with healthcare workers. These qualitative data collection activities occurred between May 6 and June 20, 2023, with follow-up validation and analysis completed in June and July 2023.

### Setting

Kiyawa Local Government Area (LGA), located in Jigawa State within Nigeria’s northwest geopolitical zone is one of the 27 LGAs in Jigawa state. The socio-cultural landscape of Kiyawa LGA is notably homogeneous, with the Hausa and Fulani ethnic groups representing 99% of residents. Agriculture serves as the primary livelihood for the majority of the population. Kiyawa LGA comprises three districts—Kiyawa, Shuwarin, and Abalago—divided into eleven electoral wards. The LGA has 11 PHCs – one per ward, and over 25 health posts that spreads across all 11 wards. Its population has grown from 172,952 in the 2006 census to an estimated 230,000 [24]. Jigawa State has one of the smallest populations in the region, with a population of 4.3 million in 2006 and a projected 7.5 million in 2022 [25, 26]. The 2024 Nigeria DHS reported that only 32.9% of children aged 12–23 months in Jigawa State were fully immunized according to the national schedule.

### Population, sampling and recruitment

#### Quantitative

##### Population

Eligible participants were women aged 16–49 years who are permanent residents of Kiyawa LGA and have at least one child aged 0–59 months and were able to present the child’s vaccination card during a household visit. We excluded women who were unavailable at the time of visit and those who refused to give consent. Children of deceased or separated caregivers were included if they remained in the same household and a caregiver was able to provide informed consent.

##### Sampling

The sampling methods have been described in previously published papers [24, 27]. We employed a multi-stage cluster sampling design, beginning with a community mapping exercise to enumerate all villages and their compounds. This process generated a complete sampling frame by recording the number of residential compounds in each village, ensuring a systematic approach to selecting potential sampling units.

For compound enumeration, we adapted the Expanded Programme on Immunization (EPI) approach [28]. First, a central point in each village was identified with the help of local gatekeepers. A random starting direction was then determined by spinning a pen, followed by sequentially numbering all residential compounds in a clockwise manner. Non-residential buildings were excluded from the count. Villages served as primary sampling units (clusters), selected through probability proportionate to size (PPS) sampling based on compound counts to ensure larger villages had a higher inclusion probability, improving representativeness. A minimum cluster size of 50 compounds was enforced to maintain statistical power. Finally, compounds were randomly selected using Stata version 16 for baseline surveys.

##### Sample size

The household survey sample size was based on the INSPIRING trial’s primary outcome, which was originally powered to detect the primary outcome through sampling of 4,480 compounds (∼127 compounds/cluster) and 9,726 children. No additional power calculations were performed as this secondary analysis relied on the trial’s established sample size [24].

#### Qualitative

To explore the factors influencing vaccine card inaccuracies, four FGDs and four IDIs were conducted. FGDs, comprising two groups of fathers and two groups of caregivers of children under five, were held in two wards (Ward 1 and Ward 2) selected based on their proximity and high reported frequency of vaccination record inaccuracies, irrespective of the percentage of inaccuracy. Community gatekeepers facilitated participant recruitment, ensuring diverse socioeconomic and demographic representation. Each FGD included 8 to 9 participants. The inclusion of fathers aimed to provide insight into household decision-making and gender-related barriers to immunization. Four FGDs were deemed sufficient for thematic saturation, given the study population’s homogeneity and focused research questions, aligning with qualitative research practices in similar settings and logistical feasibility [29].

Four IDIs were conducted with male Healthcare workers (HCWs). HCWs were selected from both FGD wards and other LGA wards using convenience sampling based on their availability. Given the specificity of the participant group and the focused nature of the inquiry, this sample size was considered adequate to generate meaningful insights [29]. The IDIs were conducted in English language by data collectors who were fluent in both Hausa and English and it aimed to capture HCW perspectives on vaccination record-keeping challenges and practices, including systemic and operational barriers contributing to inaccuracies.

### Data collection procedures

#### Quantitative

##### Compound survey

The trial endline surveys consisted of questionnaires for three respondent types in each compound: (1) compound head (2), household heads, and (3) eligible women. The compound head provided information on compound membership, structure, assets, income, and community cohesion. Women were asked about their socio-demographics, and then for each of their surviving children aged 0–59 months were requested to present their vaccination card. They were then asked to verify the accuracy of the documented information. In cases where discrepancies were noted, caregivers were asked to provide the correct vaccination history based on their recall, and a picture of the vaccine card was taken.

Data collection was conducted by 20 locally recruited female clinical data collectors, overseen by two field supervisors. Data collectors underwent a comprehensive one-month training which included practical sessions, home assignments, online webinars and a field pilot in a neighbouring LGA. The training included sessions on how to interpret vaccination cards, ask questions about maternal recall, and record discrepancies. Data were collected using a custom CommCare application on Android tablets, featuring integrated skip patterns and data cleaning rules to minimize errors.

When vaccine cards were presented, data collectors extracted key details, including vaccination dates and vaccine types (BCG, DPT-HepB-Hib, oral polio, measles), into a data collection form. Caregivers were asked to validate the card’s accuracy. In cases where caregivers reported discrepancies between the information on the vaccination card and their recollection (e.g. a vaccine documented on the card that the child did not receive), the data collector recorded the caregiver’s recollection of the vaccination details in the data collection form. To facilitate verification, the data collector then photographed the vaccine page of all presented cards, regardless of whether discrepancies were reported.

Supervisors conducted periodic spot checks and reviewed photographed card images to verify the accuracy of data entry, ensuring consistency and reliability in the data collection process. Caregivers were informed that their participation was voluntary and that their responses would be kept confidential.

##### Qualitative

Two clinical data collectors underwent a two-day training session. One collector facilitated the FGDs, while the other documented notes and posed supplementary questions. An FGD guide, informed by quantitative data findings and structured around vignettes addressing over- and under-reporting of vaccination coverage (Supplementary File S1: Vignette Scenarios used for FGDs and IDIs), was developed. These vignettes were based on real-life scenarios reported by caregivers during surveys, highlighting vaccine card inaccuracies. The HCW interview guide (Supplementary File S2: HCW interview on Discrepancies In Child Vaccine Collection) was subsequently developed using emerging FGD findings. Both FGDs and HCW interviews were conducted face-to-face, lasting 60–120 min. All FGD sessions were conducted in Hausa language, audio-recorded, transcribed, and translated from Hausa to English, while the HCW interviews were conducted in English and transcribed. Both the FGDs and the interviews were anonymized. Interviews were conducted by data collectors fluent in Hausa and English, who also performed the translations to ensure no loss of information and conceptual accuracy. The transcripts were also reviewed by another interviewer to ensure no loss of information. 

### Analysis

#### Quantitative

Descriptive statistics were used to present the sociodemographic characteristics of women and children, as well as vaccination coverage. Vaccine card documentation of “ever vaccinated” was used. Vaccine card accuracy was assessed against caregiver reports and discrepancies were flagged as inaccurate. To determine specific antigen discrepancies and their frequency, data were extracted from photographs of vaccine card records and cross-referenced with caregiver-reported vaccination status. Indecipherable information from the photographs was coded as missing data and excluded from the numerator and denominator when calculating the proportion of vaccine cards that were inaccurate.

We defined inaccuracies as discrepancies between vaccine cards and caregiver reports, classifying them into two types: over-reporting (vaccines recorded on the card but not received by the child) and under-reporting (vaccines received by the child but missing from the card). The extent of inaccuracies was quantified by calculating the proportion of children with at least one inaccuracy and the average number of inaccuracies per child. This provided a measure of the magnitude and distribution of reporting errors. Next, we stratified inaccuracies by specific antigens (BCG, DPT-HepB-Hib, oral polio, measles) and by number of doses in order to identify patterns in reporting errors. This stratification revealed whether inaccuracies were more common for certain vaccines or doses, potentially pointing to systemic issues such as stockouts, recording errors, or challenges in vaccine administration. Findings were presented in bar charts to show the proportion of inaccuracies by antigen and dose.

To determine factors associated with vaccine card inaccuracies, we conducted univariate and multivariate logistic regression analyses. The outcome variable, vaccine card inaccuracy, was binary (inaccurate = 1), and defined as any discrepancy, under-reporting or over-reporting in any recorded antigen. Explanatory variables included sociodemographic, healthcare access, educational, and financial incentive variables. The unit of analysis was the child (i.e., each child’s vaccine card) and to account for maternal-level clustering (multiple children per woman), we used mixed-effects models with random effects per woman. Variance inflation factor (VIF) tests were employed to ensure model stability by validating the absence of collinearity among predictors.

#### Qualitative

The analysis team (DB, JS, AAB and TC) conducted a conventional content analysis [30] of the interviews and FGDs using QDA Miner Lite software. DB and JS reviewed transcripts for completeness and accuracy and then independently read and coded the transcribed interviews. DB and JS then worked together to identify codes that support or contradict the reasons found in the quantitative data (Fig. 2). AAB and TC reviewed the codes and after multiple iterations of team discussion and refinement, the team agreed on the final codes which described reasons for inaccuracies in vaccine card records. Quantitative findings were integrated with qualitative results to provide context for observed inaccuracies in vaccination records.

#### Reflexivity

DB, JS and AAB are Nigerians. DB and JS hold master’s degrees in public health and were residents in Jigawa for the duration of the research project, while AAB is a community health physician and holds a PhD in Global Public Health. TC is British and a professor of global health systems, epidemiology, and evaluation. We analysed viewpoints from the perspectives of a Nigerian caregiver and a healthcare professional.

## Results

### Participant characteristics

A total of 3,232 women were included in the quantitative analysis (Supplementary Fig. 1: Participant inclusion flow diagram). Among them, 37.0% were between the ages of 16 and 24, 62.8% of the women were married before reaching the age of 16, 64.0% had at least two children under the age of 5, and only 10.4% had received formal education. Most respondents (52.4%) identified as farmers or labourers, and although 84.5% earn something, only 0.6% earned a monthly income exceeding 30,000 naira (approx. 30 US dollars) - Table 1a. Overall, 4,258 children’s vaccination cards were presented, out of the 9,726 children surveyed, from 3,232 caregivers/caregivers. Of these the 4,258 eligible children, 51.8% were male and 48.1% were 25 months and above. 441 (10.4%) of the child vaccination cards were reported to be inaccurate by the caregiver (Table 1b).

For the qualitative data, we had 33 participants across the 4 FGDs, 17 female and 16 males. The men’s ages ranged from 33 to 55 years, and women from 16 to 49 years (Supplementary Table 1: Characteristics of FGD and IDI participants).

**Table 1a:** Demographic characteristics of caregivers in Kiyawa LGA, Jigawa State

Caregiver characteristics		(*N* = 3,232)	(%)
Age group*	Less than 25 years 25–34 years 35–49 years	1,197 1,346 688	(37.0) (41.7) (21.3)
Age at first marriage	Less than 16 years 16 years and above	2,031 1,201	(62.8) (37.2)
Highest Level of Education**	None Informal/religious/formal	861 2,368	(26.7) (73.3)
Marital status	Married Others	3,219 13	(99.6) (0.4)
Religion	Islam Others	3,229 3	(99.9) (0.1)
Number of surviving children	Less than 3 3–4 children 5 children and above	943 1,078 1,211	(29.2) (33.4) (37.5)
Number of under-5 children	1 2 and above	1,165 2,067	(36.0) (64.0)
Occupation	Not working/Housewife Farming/Manual Labour Business/Professional/TBA	567 1,693 972	(17.5) (52.4) (30.1)
Actual Income***	Nothing < 30,000 naira ≥ 30,000 naira	497 2,700 19	(15.5) (83.9) (0.6)
Marriage type****	Only wife More than one	1,579 1,639	(49.1) (50.9)
**Characteristics of care-seeking facilities according to women**		**(*N = 3***,**232)**	**(%)**
Main Careseeking facility¤	PHC	2,722	(87.2)
	Secondary	325	(10.4)
	Tertiary	76	(2.4)
Number of facility’s visit last 3 months	0	381	(11.8)
	1 or 2	2,622	(81.1)
	≥ 3	229	(7.1)

*Missing age (*n* = 1); **Missing education (*n* = 3); ***Missing monthly income (*n* = 16); ****Missing marital status (*n* = 14), ¤Missing care facility (*n* = 109); all other variables had no missing data; TBA= Traditional Birth Attendant

**Table 1b:** Demographic characteristics of children in Kiyawa LGA, Jigawa State

Characteristics of children whose cards were presented by caregivers		(*N* = 4,258)	(%)
Child’s sex	Male Female	2,206 2,052	(51.8) (48.2)
Child’s age	0–11 months 12–24 months 25 months and above	1,181 1,030 2,047	(27.7) (24.2) (48.1)
Multiple birth	Singleton Multiple	4,138 120	(97.2) (2.8)
Child sick in the last two weeks	Yes No Don’t know	884 3,370 4	20.8 79.1 0.1
Has the caregiver confirmed that the vaccine card is accurate?	Not accurate Accurate Missing	441 3,609 208	(10.4) (84.7) (4.9)

### Vaccine coverage among under 5 children, based on information provided by the caregivers

Analysis of immunization records (Table 2) revealed high reported coverage for most basic vaccines among the 4,258 children with available vaccination cards. Nearly all caregiver’s recall and card information indicated receipt of BCG (caregiver’s recall = 98.3%; card information = 98.3%), polio-0 (caregiver’s recall = 99.1%; card information = 98.2%), and Penta-1 (caregiver’s recall = 93.1%; card information = 93.2%), while measles 2 vaccination rates were notably lower (caregiver’s recall = 17.7%; card information = 17.6%). Maternal recall data showed similar coverage patterns to card records, with minimal discrepancies between the two sources. The agreement between maternal recall and card information was high, exceeding 99% concordance for all vaccines assessed.

**Table 2:** Comparison of vaccine coverage estimates from vaccination cards and maternal recall among children under 5 in Kiyawa LGA (*N* = 4258)

Vaccine type	Caregiver’s recall	% recall coverage	Card information	% card coverage	Discrepancy between caregiver’s recall and card coverage %
BCG	4186	98.3	4186	98.3	0.0
POLIO 0	4220	99.1	4180	98.2	-0.9
POLIO 1	3930	92.3	3945	92.6	0.4
POLIO 2	3661	86.0	3668	86.1	0.2
POLIO 3	3226	75.8	3232	75.9	0.1
PCV 1	3939	92.5	3953	92.8	0.3
PCV 2	3681	86.4	3682	86.5	0.0
PCV 3	3374	79.2	3369	79.1	-0.1
PENTA 1	3963	93.1	3968	93.2	0.1
PENTA 2	3697	86.8	3695	86.8	0.0
PENTA 3	3384	79.5	3379	79.4	-0.1
MEASLES 1	2600	61.1	2590	60.8	-0.2
MEASLES 2	754	17.7	748	17.6	-0.1

### Discrepancies by wards

Table 3 presents the prevalence of inaccuracies in vaccine cards across different wards, revealing that while the majority of cards were accurate (84.8% overall), there was notable variation by ward: Kiyawa (97.3%) and Kwanda (97.7%) exhibited the highest proportions of accurate cards, while Garko (15.5%), Katanga (13.1%), Katuka (14.6%), and Shuwarin (15.1%) showed the highest percentages of inaccurate cards. The proportion of respondents who were “not sure” about the accuracy of their cards also varied, with Balago (11.5%) and Tsirma (14.8%) having the highest percentages in this category.

**Table 3:** Caregiver perception of vaccine card accuracy by ward (*n* = 4 258 cards)

Wards	Not accurate	% Not accurate	Accurate	% Accurate	Not sure	% Not sure	Total	% Total (row)
Andaza	34	8.2	353	85.3	27	6.5	414	100
Balago	7	1.8	333	86.7	44	11.5	384	100
Fake	75	10.5	603	84.7	34	4.8	712	100
Garko*	50	15.5	264	82	8	2.5	322	100
Katanga	60	13.1	377	82.1	22	4.8	459	100
Katuka*	81	14.6	448	80.6	27	4.9	556	100
Kiyawa	2	2.7	72	97.3	0	0	74	100
Kwanda	4	1.8	215	97.7	1	0.5	220	100
Maje	13	6	204	94	0	0	217	100
Shuwarin	96	15.1	534	84	6	0.9	636	100
Tsirma	19	7.2	206	78	39	14.8	264	100
Total	441	10.4	3,609.0	84.8	208	4.9	4,258.0	100

*Selected for the formation of the FGD groups

### Discrepancies by vaccine

**Fig. 1:**
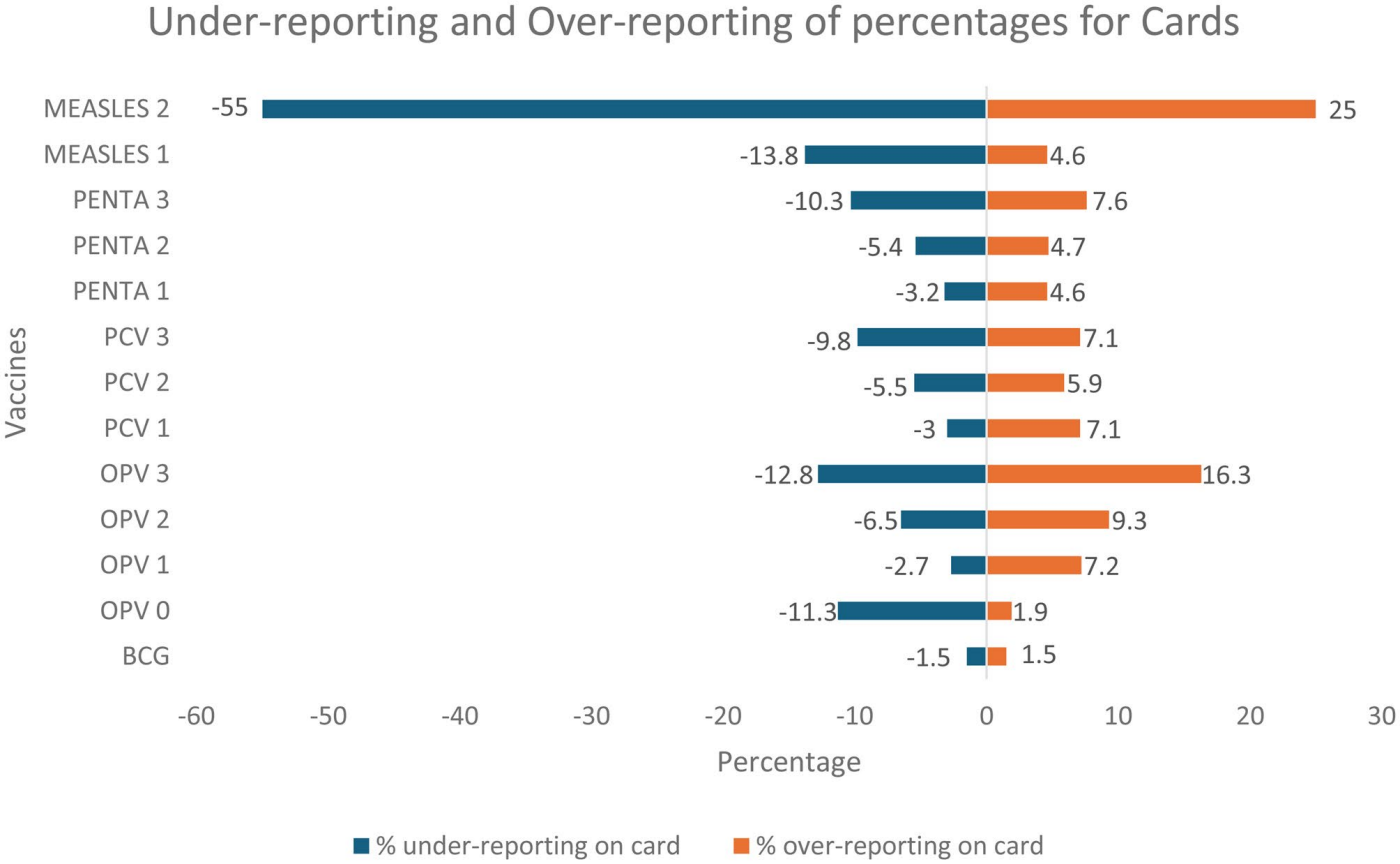
Percentage over-reporting and under-reporting for caregivers who reported vaccine card inaccuracy

Analysis of vaccination recording accuracy revealed distinct patterns across vaccine types (Table 4; Fig. 1). BCG demonstrated the highest data consistency between cards and maternal recall, with minimal under-reporting (1.5%) and over-reporting (1.5%). For the Polio series, recording discrepancies showed more for two doses - OPV0 and OPV3 showed greater discrepancies of under-reporting (OPV0 = 11.3%; OPV3 = 12.8) and OPV3 showed substantial over-reporting (16.3%). The PCV and PENTA series exhibited moderate discrepancies, with slightly higher under-reporting for later doses. Most notably, Measles-2 vaccination showed the poorest agreement between sources, with a striking under-reporting (55.0%) and substantial over-reporting (25.0%) on cards, suggesting this vaccine was both frequently missed in practice and inconsistently documented when administered.

**Table 4:** Differences in caregiver’s recall and card details of children whose caregivers reported inaccuracy by vaccine, using the caregiver’s recall as reference (*N* = 439)

	Caregiver’s recall	Card details	Under reporting on card (Recall not Card)	Over reporting on card (Card not Recall)	Card extra	% under reporting on card	% over reporting on card
BCG	409	409	6	6	0	1.5	1.5
Polio 0	423	383	48	8	-40	11.3	1.9
Polio 1	334	349	9	24	15	2.7	7.2
Polio 2	247	254	16	23	7	6.5	9.3
Polio 3	172	178	22	28	6	12.8	16.3
PCV 1	337	351	10	24	14	3.0	7.1
PCV 2	253	254	14	15	1	5.5	5.9
PCV 3	183	178	18	13	-5	9.8	7.1
PENTA 1	345	350	11	16	5	3.2	4.6
PENTA 2	257	255	14	12	-2	5.4	4.7
PENTA 3	184	179	19	14	-5	10.3	7.6
MEASLES 1	109	99	15	5	-10	13.8	4.6
MEASLES 2	20	14	11	5	-6	55.0	25.0
Total			213	193			
Average			213/439 = 0.49	193/439 = 0.44			

*2 cards have no record

### Factors associated with inaccuracy reports by caregivers

The adjusted analysis found that a woman’s educational status, having a co-wife and child age were all significantly associated with reporting card inaccuracies. Women with any form of education had higher odds of reporting inaccuracies (OR = 1.33, 95% CI [1.03, 1.75], *p* = 0.032); having a co-wife was associated with a decreased odds of recording inaccuracies (OR = 0.78, 95% CI [0.62, 0.98], *p* = 0.038); while there was also higher odds of reporting inaccuracies for older children (12–24 months-OR = 2.70, 95% CI [1.94, 3.75], and 25 months and above-OR = 2.30, 95% CI [1.69, 3.12], both *p* < 0.001) compared to children under 1 year. Only health status of child in the last 2 weeks (OR = 1.39, 95% CI [0.98, 1.59], *p* = 0.005) and facility where care was sought (OR = 1.92, 95% CI [0.84, 2.65], *p* = 0.017) was significant in the unadjusted analysis but not significant in the adjusted analysis (Table 5).

Caregiver’s age and age at first marriage, the number of children alive and number of under-five children, caregiver’s occupation, caregiver’s income, child birth order were not significantly associated with reporting card inaccuracies (Table 5).

**Table 5:** Multivariable logistic regression analysis of any (under or over) inaccuracies in child vaccination cards, adjusted for maternal-level clustering

	Outcome variable: child vaccine card inaccurate (under- or over-reporting of any antigen)							
	Unadjusted (each variable considered separately)				Adjusted (all variables included in one multivariable model)			
Variables	Odds ratio	95% confidence interval		*p*-value	Odds ratio	95% confidence interval		*p*-value
**Caregiver’s age**								
Less than 25 years	ref				Ref			
25–34 years	0.91	(0.71	1.16)	0.449	0.95	(0.70	1.29)	0.724
35 years and above	1.09	(0.83	1.45)	0.511	1.05	(0.72	1.53)	0.816
**Age at first marriage**								
Less than 16 years	ref				Ref			
16 years and above	1.14	(0.92	1.42)	0.232	1.13	(0.91	1.43)	0.263
**Level of education**								
None	ref				Ref			
Any education	1.34	(1.05	1.74)	**0.021**	1.33	(1.03	1.75)	**0.032**
**Number of children alive**								
Less than 3	ref				Ref			
3–4	0.80	(0.61	1.05)	0.104	0.87	(0.63	1.21)	0.417
5 or more	0.95	(0.74	1.23)	0.705	1.03	(0.71	1.49)	0.888
**Number of under-5 children**								
One	ref				Ref			
2 or more	0.81	(0.65	1.00)	0.053	0.87	(0.68	1.10)	0.259
**Occupation**								
Not working/Housewife	ref				Ref			
Farming/Manual labour	0.84	(0.62	1.15)	0.286	1.38	(0.68	2.80)	0.373
Business/ professional/TBA	1.11	(0.81	1.55)	0.503	1.78	(0.86	3.70)	0.122
**Earn Income**								
Earn nothing	ref				Ref			
Earn something	0.80	(0.59	1.08)	0.147	0.51	(0.25	1.02)	0.056
**Have co-wife**								
None/Only wife	ref				Ref			
More than one	0.76	(0.61	0.94)	**0.010**	0.78	(0.62	0.98)	**0.038**
**Child birth-order**								
Singleton	ref				Ref			
Multiple	1.10	(0.52	2.35)	0.795	1.11	(0.48	2.61)	0.802
**Child’s age**								
Less than 1 year	ref				Ref			
12–24 months	2.74	(2.00	3.75)	**< 0.001**	2.70	(1.94	3.75)	**< 0.001**
25 months and above	2.27	(1.70	3.04)	**< 0.001**	2.30	(1.69	3.12)	**< 0.001**
**Morbidity (sick in the last 2 weeks)**								
No	ref				Ref			
Yes	1.39	(1.11	1.74)	**0.005**	1.25	(0.98	1.59)	0.065
**Care seeking facility type**								
PHC	ref				Ref			
Secondary	0.82	(0.57	1.18)	0.290	0.79	(0.55	1.15)	0.219
Tertiary	1.92	(1.12	3.29)	**0.017**	1.49	(0.84	2.65)	0.170

### Reasons for inaccuracy of child vaccination card according to caregivers’ response

#### Lack of knowledge of vaccination schedule or missed vaccination

**Fig. 2:**
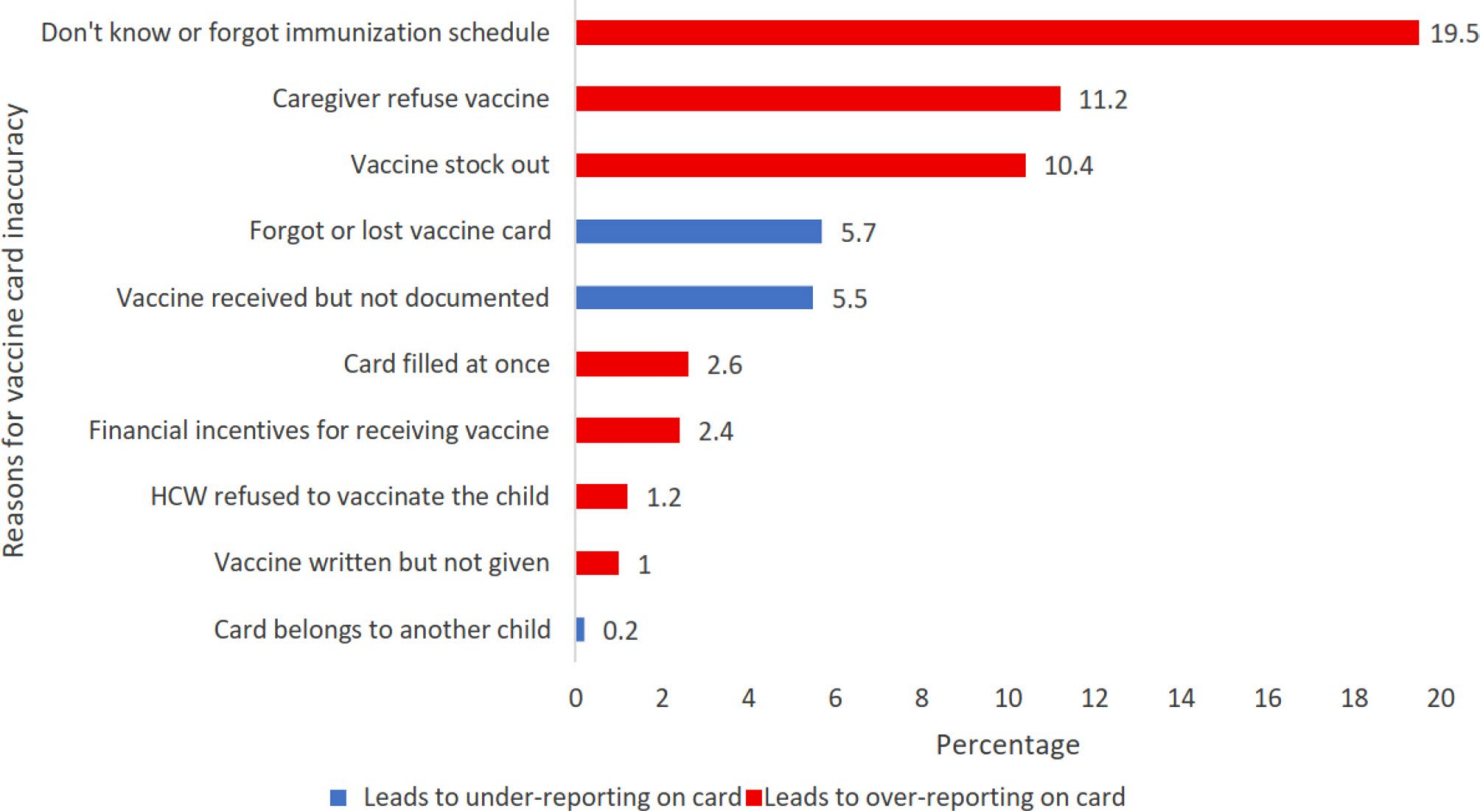
Caregiver-reported reasons for inaccuracies in child vaccination cards (Multiple responses allowed; *N* = 441)

Among 441 caregivers reporting inaccuracies in their child’s vaccination cards, the most common reason cited was maternal lack of knowledge of vaccination schedule or missed vaccination (19.5%; Fig. 2). Missed vaccinations as a result of caregiver being too busy, schedule forgetfulness or child being sick on the vaccination day, gave room for healthcare workers to completely fill the child’s vaccination card during follow-up visits, even if the missed dose had not been administered, thus leading to over-reporting.

*Number one thing is illiteracy most of women here don’t go to school for Western education and don’t go out that much to mingle with people of different cultures or practise and may not know when vaccines are due* ***(HCW 01)***

*This type of situation happened before, there was one healthcare worker some years ago I can’t remember the year but that time supervisors use to come for routine immunization supervision. So, one day a supervisor came and the healthcare worker came to our houses and collected our children vaccines card and filled for those that are not up to date according to their age and the parent were told to answer yes if the supervisor asked them whether they children were vaccinated up to date****(Participant 6***,*** Male***,*** Ward 02)***

*The in-charge told me to go with him to do our quality survey before the supervisor comes. So, we used 2 days and visited all the communities under the ward to check their cards and we corrected any card we see error. We fill in any cards that is not fully immunized by age or by antigen according to the cards… The supervisor came the 3rd day and did the quality survey, and we passed greatly.****(HCW 01)***

#### Caregiver refused vaccination

Vaccine refusal by caregivers (11.2%) emerged as another significant contributor to vaccination card inaccuracies in our quantitative findings (Fig. 2). Qualitative data corroborated this observation, identifying vaccine hesitancy as a key underlying factor. In some cases, hesitant caregivers permitted healthcare workers to complete their child's vaccination card despite non-administration of vaccines, primarily to avoid subsequent clinic visits, leading to over-reporting of vaccine received. This practice reflected both caregivers' desire to limit health system engagement and healthcare workers' reluctance to make repeat visits to remote villages with challenging accessibility due to poor road conditions. These findings may also account for the 2.6% of caregivers who reported their child's vaccine card was filled all at once during a single visit (Fig. 2).

*The government is not confronting this issue, because how can LGA team tell me to fill cards for defaulters knowing that they rejected the vaccine but I don’t have choice than to just do as I was told. Because if I don’t obey them, they will threaten me* ***(HCW 04)***

*There are households that don’t accept immunization in this community the healthcare worker knows them, so the healthcare worker use to fill immunization card for their children and not immunize the children ****(Participant 8***,*** Male***,*** Ward 01)***

#### Healthcare workers refuse to vaccinate the children

Similarly to caregiver’s vaccine hesitancy, our qualitative findings further elucidate the relationship between vaccination card inaccuracies and hesitancy among healthcare workers themselves who refuse to vaccinate the children (1.2%), leading to over-reporting. This phenomenon can directly compromise vaccination program integrity, as hesitant HCWs may complete vaccination cards without administering the actual vaccines. Such practices not only create inaccurate immunization records that may leave individuals unprotected but also erode community trust in vaccination programs. These concerning findings were corroborated by reports from both healthcare workers and community members.

*Some of us healthcare workers we don’t believe in this vaccines things oooo*,* there is one of my close friend I would not mentioned his name. But he is a community health extension worker and he is providing Immunization services but he doesn’t give his children the immunization. He only fill card for them. He has two children now but all of them has never been immunized* ***(HCW 01)***

*Some healthcare workers were the ones discouraging people…So if healthcare worker who is supposed to encourage people are discouraging them, what do you expect from a common man who only act on instruction of healthcare workers****(Participant 4***,*** Male***,*** Ward 02)***

#### Vaccine and vaccine card stockouts

Our findings also identified systemic challenges contributing to vaccination card inaccuracies. Among caregivers, 10.4% reported vaccine stockouts during immunization sessions, while 5.7% attributed discrepancies to lost or forgotten cards, and 5.5% indicated their child received vaccines that were never documented (Fig. 2). These logistical barriers frequently lead to inaccurate record-keeping. Qualitative data also revealed that healthcare workers often improvise with temporary paper records when facing vaccine card stockouts or when caregivers present without their vaccination cards. Although well-intentioned, these stopgap measures meant to preserve immunization histories often fail when temporary records aren’t transferred to official cards, leading to under-reporting. Both healthcare workers and community members acknowledged this persistent documentation gap, underscoring the vulnerabilities in maintaining reliable vaccination records in resource constraints settings.

* If a caregiver forgot the child’s immunization card at home and if it is within community where the healthcare center is located she or he would be asked to go back home and bring the card before the child would be immunized*,* but if they are far from the healthcare centre*,* the healthcare worker would immunized the children and record the vaccine given to the child on a sheet of paper*,* which can easily be misplaced ****(Participant 4***,*** Male***,*** Ward 02)***

*The healthcare worker told me there was no vaccines card available, but he used white sheet of paper and enter my baby’s information and he also entered it into the immunization register* ***(Participant 1***,*** Female***,*** Ward 02)***

*What we normally do whenever we don’t have vaccine cards is, I used to buy exercise book and then cut pieces of paper from it then use it for new visit and for those that lost their vaccine card* ***(HCW 03)***

In some instances, as reported by a caregiver, even temporary paper documentation may be omitted entirely.

*For me I thought that, may be that was how the immunization of that day should be. To vaccinate the children without documenting ****(Participant 7***,*** Female***,*** Ward 02)***

#### Filled in vaccine card before administering vaccines

Additionally, some vaccinators pre-emptively complete vaccination cards for all children that came to the facility for immunization before administering vaccines. Such practice that leads to inaccuracies when vaccine stockouts occur (10.4%) or caregivers leave before their child’s turn to receive the vaccine, leading to over-reporting. Inaccuracies are further compounded when documentation and vaccine administration are handled by separate healthcare workers, creating mismatches between records and actual vaccinations, and undermining the reliability of immunization data.

*The scenario I witness was that the immunization card was filled but the vaccines got finished so the caregiver was asked to come back the following week to receive the vaccines, which they don’t usually do ****(Participant 7***,*** Female***,*** Ward 01)***

*Scenarios like this use to happen where the health care workers would fill the immunization card and register before administering the vaccines so in some cases the vaccines might finish and some children will not be immunized ****(Participant 2***,*** Female***,*** Ward 01)***

#### Financial incentives for receiving vaccines

Financial incentives for receiving vaccines, just like in the quantitative study (2.4%), also emerged as a potential concern in the qualitative study. In this setting where immunization is incentivized, caregivers may prioritize obtaining these incentives over accurate record-keeping, potentially leading to misuse of vaccine cards such as duplicate cards or borrowing another child’s documentation. Healthcare workers and caregivers alike reported these practices, underscoring how well-intentioned incentive programs can inadvertently compromise data integrity.

*I had one child that died who is older than the one alive and his immunization card was up-to-date so we concluded to give the supervisor the deceased child card for the one alive and that was what we did. He was so happy to see the card all up to date and he gave us 2,000 naira that day* ***(Participant 2***,*** Male***,*** Ward 02)***

*Now as it is we don’t even know those whose card has actually been lost… Some women would take their children for the same immunization in different hospitals especially measle because they would receive 2,000 naira as incentives. You would hear a woman narrating beautiful lies just to receive 2,000 naira for 5th visit especially Measles vaccine****(HCW 02)***

This statement by HCW 02 further explains why discrepancies is much higher for measles 2 vaccine compared to other vaccines, as shown in fig. 1.

In addition, the absence of a centralized digital immunization registry further exacerbates documentation discrepancies. In the current fragmented system, healthcare workers cannot verify a child’s complete vaccination history when families relocate, particularly if the physical vaccine card is missing. For instance, when new residents present without documentation, providers must issue a new card that only captures vaccinations administered at their facility—resulting in incomplete records that compromise accurate immunization tracking and follow-up.

*He told me that I have to go back to Bauchi for them to fill the first and second dose. Because he doesn’t know the exact date and I don’t know myself. That was how I completed the remaining immunization without filling the first two. The card is still like that half-filled till now because I have not gone back to Bauchi****(Participant 7***,*** Female***,*** Ward 01)***

Other reasons we identified in the qualitative data include:

#### Poor road infrastructure

Healthcare workers also highlighted significant logistical challenges in reaching remote settlements for outreach programs, primarily due to poor road infrastructure caused by poorly planned facility locations. The lack of motor vehicle access to these areas complicates outreach sessions and delays the delivery of essential vaccines. This not only impedes immunization efforts but also exposes healthcare workers to travel-related accidents. Additionally, such accidents risk the loss of vital antigens and vaccine cards, further jeopardizing public health initiatives.

When such accidents occur and antigens or vaccine cards are lost or damaged, healthcare workers may resort to either administering vaccines without documenting them or recording doses without actual administration, compromising both accountability and immunization coverage.

*There are communities that if not because of this work I wouldn’t go there till I die. There was a day I was going to Barka community for outreach*,* I fell from the bike and the vaccination box also fell and some of the antigens broke. Similar thing happened to my friend last year during raining season when he was going to one of the communities in Kwanda ward. he fell inside the river with the vaccination box****(HCW 01)***

#### Missed opportunity

Another key concern was the perceived “missed opportunity” when a child could not be vaccinated due to a lost vaccination card. In such cases, driven by the public health imperative to vaccinate, HCWs sometimes administered the vaccine even without the card, a practice corroborated by caregiver testimonies during focus group discussions.

*It is better to immunize a child without recording it than to record without giving the immunization because the most important thing is immunizing the child. In fact, that is exactly what our health care worker are doing in this area****(Participant 1***,*** Female***,*** Ward 01)***

## Discussion

Our study assessed prevalence of inaccuracies in vaccine cards record and explored underlying causes for discrepancies in vaccine card documentation. Our quantitative analysis showed that there are positive correlations between a woman’s education, having a co-wife, child’s age, and reporting of inaccuracy in their child’s vaccination card, while the qualitative context provided by the FGDs and interviews sheds more light on the factors contributing to discrepancies between caregiver reports and vaccine card records. We observed that the reasons for inaccuracies in child’s vaccination card could be attributable to caregivers, HCWs, and the influence of health system factors on immunization service delivery [31].

Our analysis reveals that maternal education level significantly influences the detection of discrepancies in vaccination records. Literate caregivers, equipped with skills to critically evaluate immunization cards, demonstrated greater capacity to identify inconsistencies between documented and administered vaccines [32]. This increased awareness stems not only from an ability to interpret card information but also from a stronger understanding of the card’s role in tracking immunization history. Such caregivers were more likely to engage proactively with healthcare workers regarding irregularities, thereby serving as a corrective mechanism for record-keeping errors [32, 33]. However, while education enhances discrepancy detection, it represents just one factor among many contributing to vaccination record inaccuracies in Jigawa’s complex immunization ecosystem.

The perceived or actual inaccuracies of vaccine cards from older children may be because older children may have experienced changes in caregivers or may have accessed immunization from different facilities, leading to fragmented records [34]. However, this finding may be multifactoral, also stemming from time related card retention to caregiver recall challenges [12]. These challenges underscore the need for an electronic health records system and caregiver education on the importance of long-term card retention.

Our analysis also found that women with co-wives are more likely to report vaccine card inaccuracy compared to women who are sole wives of their husband. Women in polygamous homes may have greater support networks, more shared experiences, and increased social pressure to demonstrate good mothering, all of which encourages retention and use of vaccine cards [35].

Furthermore, our study identifies vaccine card stockouts as a critical driver of reporting inaccuracies in Jigawa’s immunization system. During shortages, healthcare workers (HCWs) often resort to paper substitutes, which are prone to loss or damage before subsequent visits. Compounding this issue, caregivers whose children complete their vaccination schedule during stockouts rarely return to transfer paper records to official cards once supplies are replenished. This gap systematically undermines data reliability. Miles et al. 2013, provides a broad evidence base showing that whenever the vaccination card is absent and substitutes are used, coverage estimates and program indicators may be biased [36]. Wagner et al. 2019 further highlights how cards serve as vital clinical decision-making tools by documenting vaccination histories [37]. While electronic systems (common in high-income countries) and low-cost alternatives like vaccine bracelets [38–40] show promise, paper-based records remain essential in low-resource settings, particularly for mobile populations who may transition between different health systems. These findings underscore the urgent need to stabilize vaccine card supply chains to preserve data integrity in immunization programs.

Meanwhile, our study also reveals how vaccine misinformation fuels caregiver hesitancy, directly contributing to inaccuracies in immunization records [41]. Despite refusing vaccines, some caregivers nonetheless request completed vaccination cards, suggesting an attempt to avoid subsequent visits from HCWs [42, 43] or gain associated incentives [44]. Furthermore, pressure on health workers to achieve high coverage metrics may inadvertently encourage this practice, which not only distorts data integrity but also risks further alienating caregivers, creating a vicious cycle of distrust and unreliable record-keeping. This necessitates robust monitoring and evaluation (M&E) systems capable of detecting both data discrepancies and emerging trust issues among caregivers and health workers [45].

Findings from our HCW interview revealed critical staffing shortages exacerbate these documentation challenges, with HCW reporting unsustainable patient loads during immunization sessions. These overwhelming conditions directly contribute to record-keeping lapses, including missed card entries and omitted health education components. Existing research corroborates that such staffing constraints limit service quality and completeness [4, 9, 46], particularly in documentation practices. Addressing these personnel gaps must become an immediate priority, as accurate record-keeping fundamentally depends on having adequate, well-supported health workers to maintain proper procedures during vaccination sessions.

Financial incentives designed to boost vaccination rates [47] may inadvertently compromise documentation accuracy and child safety. Our interviews revealed that caregivers facing economic hardship sometimes seek unnecessary vaccinations to obtain incentives, particularly for higher-value vaccines like measles-2, where incentive-linked discrepancies were most pronounced. This aligns with findings by Bakare et al. [48] and Moro et al. [49] on financial incentives driving excess vaccination. Additionally, campaign-based delivery of certain vaccines (e.g., polio [50, 51]) further complicates accurate record-keeping. These findings underscore how incentive structures can distort both vaccination practices and documentation. Implementing biometric-linked electronic systems could address these issues by maintaining reliable vaccination histories, preventing unnecessary doses [52], and eliminating card-related access barriers that currently compromise data integrity.

### Limitations of the study

Our study has two key limitations. First, including all children under five years of age may introduce recall bias for vaccinations administered more than one year before the study, particularly for children ≥ 12 months (see Supplementary Table 2) [53]. Second, when discrepancies occurred between vaccination cards and caregiver reports, we prioritized caregiver responses if card information appeared incomplete or inconsistent. However, we recognize that caregiver recall is not a definitive gold standard. Caregiver memory is vulnerable to recall bias, which varies by time elapsed, vaccine type, and social desirability bias, which may lead to under-reporting or over-reporting. Therefore, observed discordance may reflect errors in caregiver memory rather than documentation failure alone, potentially affecting the accuracy of the findings. The reliance of the quantitative component on maternal reports may reduce comparability with the qualitative findings. While maternal recall served as a pragmatic comparator for assessing vaccination card inaccuracies, its inherent limitations, such as potential memory errors or incomplete knowledge of immunization schedules, are acknowledged. Nevertheless, this approach aligns with the study’s objective of evaluating card accuracy in a setting without electronic immunization registries.

## Conclusion

This mixed-methods study reveals that the inaccuracies in Jigawa’s immunization records stem from interconnected health system weaknesses (particularly fragmented documentation practices) and caregiver-level factors (including vaccine hesitancy and limited health literacy). While financial incentives can improve vaccination rates, our findings caution against implementation strategies that prioritize documentation compliance over actual vaccine administration. Immediate solutions could combine innovations like vaccine bracelets to supplement paper records, SMS-based appointment reminders to reduce missed opportunities, and mandatory healthcare worker verification of immunization history during each visit, even as cloud-based digital systems remain the optimal long-term solution for comprehensive record-keeping. Future studies should employ multi-source data triangulation (e.g., facility registers, community health records and surveys) to enhance the validity of coverage assessments and better evaluate documentation accuracy in low-resource settings.

## Supplementary Information

Below is the link to the electronic supplementary material.

Supplementary Material 1

## Data Availability

Data will be made available on request.

## Abbreviations

LMICs: Low and middle–income countries
DTP: Diphtheria tetanus–pertussis
DHS: Demographic and Health Surveys
NDHS: Nigeria Demographic and Health Survey
SDG: Sustainable Development Goal
LGA: Local Government Area
FGDs: Focus Group discussions
IDIs: In depth interviews
EPI: Expanded Programme on Immunization
PPS: Probability proportionate to size
VIF: Variance inflation factor
HCWs: Healthcare workers
