# Supplementary material for: Uncovering and investigating reasons for inaccuracies in vaccine card records in Kiyawa LGA Jigawa, Nigeria: a mixed method study
Source: BMC Health Serv Res. 2026 Jun 6;26:1030. doi: 10.1186/s12913-026-14826-2 (PMC13411141; doi:10.1186/s12913-026-14826-2)
Supplement: Supplementary file 1 — Supplementary Material 1 [file 12913_2026_14826_MOESM1_ESM.docx]

**Supplementary Figure 1: Participant inclusion flow diagram**


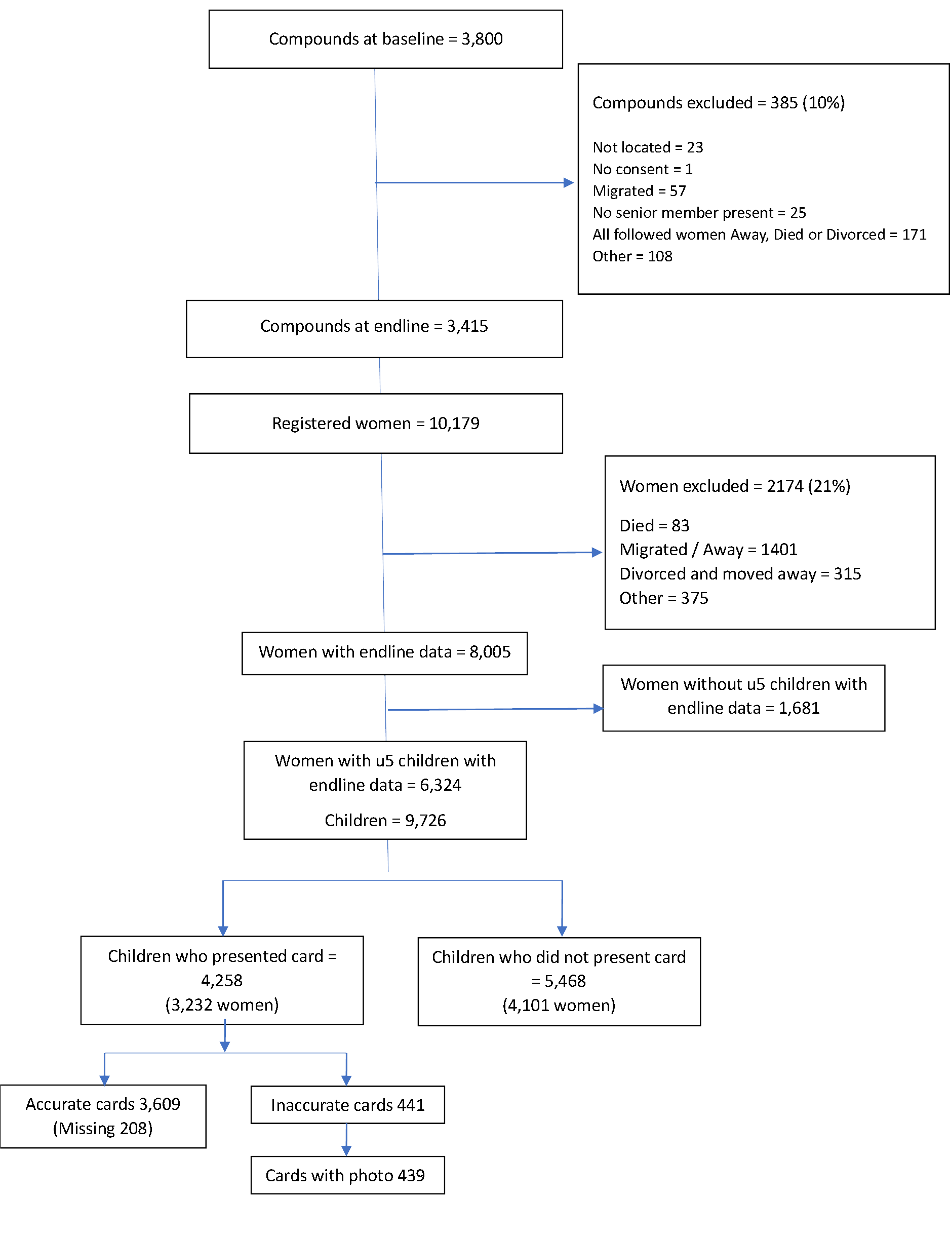


**Supplementary File S1: Vignette Scenarios used for FGDs and IDIs**

*Note – all names and case scenarios were made up*

Good morning everyone, thank you for agreeing to participate in the activity. We will be discussing some issues, many of which can be easily denied but are events we have seen ourselves and have discovered are going on in several communities around us. We will like to hear your thoughts on why these things are happening so we can collectively think of a way to solve the problems.

1. A certain Nurse Hadiza once went to the Masaya community, close to Katuka to vaccinate children. When she arrived at Mallam Abdullahi's compound, she met Mariam, Mallam Abdullahi's wife, who has two children named Isah and Musa. Isah, who is five years old, has stopped collecting child vaccines, but Musa was due for one. The nurse asked for Musa's card, but Mariam couldn't find it; instead, she found the card for the older son (Isah) and gave the Nurse. Musa was vaccinated by the Nurse and his vaccination was recorded in his brother’s card. I will like you to take your time to reflect and this and tell us similar occurrences that you have witnessed in our locality.
   1. What could have been responsible for this kind of action happening around us?
   2. What did the health care workers do in similar situation like this.? **(recycling older children’s cards)**
2. Nurse Sakina was conducting a vaccination outreach in the Sabon Gari community of Katanga. When she arrived at Mallam Hussaini's compound, she vaccinated the children without asking for their vaccine cards
   1. Tell us about similar situations like this that has happened in your community or a nearby community and what you did when that happened.
   2. What is your opinion about this practice? **(Vaccinated without documenting)**
3. Farida Ishaqu brought her twin sons, Hassan and Hussaini, to Gwazaye Hospital in Dumbal, but upon arrival, she realized she had left Hassan's immunization cards at home. Although the nurse on duty vaccinated both children, she recorded the vaccinations on Hussaini's card.
   1. We have seen this happen very well, so what do you do when this happens and what steps have the nurses been taking? **(multi persons on a single vaccination card)**
4. Nurse Fatima, who was dispatched by her hospital supervisor to provide vaccinations to children in Gidan Tsika, a community located in Shuwarin, instead collected the children's cards from the mothers and proceeded to write on them without administering any vaccines. She then departed the community without providing any vaccination services to the children.
   1. What do you do when you see Nurses doing something similar**(documented without vaccinating)**
   2. What was the nurses explanation when you asked what they were trying to do
5. In Fake, a woman gave birth to twin girls, Maida and Maimuna, at a hospital where there was no child vaccine card available. However, Nurse Quadri, one of the nurses on duty went ahead and vaccinated the babies and then recorded the details on a piece of paper which they gave to the mother.  **(substitution in case of card stock out)**
   1. We heard similar things have been happening here, what do you as parents do when there is no card available and what do the Nurses do themselves.
6. Once upon a time in a small village near Maje town, there lived a mother with two young children, Kabiru and Kamoru, who were both three years old and the same gender. Unfortunately, one of the children fell ill with a fever and passed away. The deceased child had a more complete vaccination card than their surviving sibling. When healthcare workers visited the village, they offered some money as a reward for children with up-to-date vaccine records. The mother was then faced with a difficult decision: use the vaccination card of the surviving child, which was not up-to-date, or use the more complete vaccination card of the child who had passed away.
   1. What do mothers in this community do in this type of situation. **(Using a dead child's vaccine card for a living child). Why?**

**Supplementary File S2: HCW interview on Discrepancies In Child Vaccine Collection**

We are seeking to understand vaccine discrepancies on child immunization cards among under 5 children in Kiyawa LGA. You are being invited to take part in this study as you are a healthcare provider who provides immunization services in Kiyawa LGA of Jigawa state, Nigeria. We have heard opinions of your fellow healthcare workers on this topic and have decided to involve you because of your expertise on immunization services.

**Familiarity Section**

1. Can you please tell us about yourself
   1. Your age
   2. How long you have been working here
   3. Your role in this facility, your involvement in immunization activities.

**Challenges**

1. What challenges do health care workers face during routine immunization (facility/outreach).
   1. Personal challenges
   2. Challenges caused by fellow HCWs/colleagues
   3. Challenges caused by caregivers themselves
   4. Challenges cause by religious leaders
   5. challenges from government and other agencies involved in Health care
   6. What are those specific challenges faced by HCW or caregivers during immunization, either at the facilities or during outreach

**Issues**

1. We have seen that some HCWs vaccinate children without filling their cards. ***(remember to tell them they have been selected because of their expertise, so we expect to get enough feedback from them)***
   1. What is your opinion on this
   2. What do you think is responsible for this
   3. Can you tell us your personal experience on this act
   4. How do you think this problem can be resolved
2. During our survey, we also found that some HCW fill cards without giving the vaccines.
   1. What is your opinion on this
   2. What can be responsible for this practice
   3. Can you please tell us your personal experience or that of a colleague on this practice
   4. How can this problem be resolved
3. We have heard care giver say their child immunization is recorded on a sheet of paper. ***(also talk about women who just moved in from another settlement but came without their child’s immunization card)***
   1. How true is this and what is your opinion concerning this act
   2. What could be responsible for this*.* ***(remember to ask if they track women who have been giving a paper for temporary use for proper documentation)***
   3. Can you please share your personal experience
   4. Are there other substitutes asides pieces of paper
   5. What should be done to address this issue
4. We have seen in some communities that women present immunization cards of their older children to be used for their younger ones, and the HCW workers go ahead to immunize the child and fill the card.
   1. What is your opinion concerning this?
   2. Can you please share your personal experience or that of a colleague? ***(Share how you address similar issues).***
5. During our sample, we also found some twin children share a single immunization card. ***(chip in that some share for some vaccines while others share for all vaccine received)***
   1. What is your opinion on this?
   2. Why do you think this is happening?
   3. Can you please share your personal experience or that of a colleague concerning similar occurrence?
6. Some women have admitted they will gladly cheat the system on immunization days/ outreach days in other to qualify for incentives.
   1. some have claimed they have given their child’s immunization cards to other women so they can get incentives as well
   2. some also claimed they have presented completely filled immunization cards of an older dead child for a younger child
   3. Some also admitted

***(only mention this examples after they have freely talked about other events and have nothing more to say. This examples can serve as a reminder for them)***

**Supplementary Table 1: Characteristics of FGD and IDI participants**

| **Characteristics of participants for the FGD** | | | |
| --- | --- | --- | --- |
|  |  | Men (n=16) | Women (n=17) |
| Age |  | 33 to 55 | 16 to 49 |
| Occupation | Trader | 1 | 6 |
|  | Skilled Manual Labour | 3 | 4 |
|  | Farmer | 5 | 2 |
|  | Unskilled Manual Labour | 4 | 3 |
|  | Civil servant | 2 | 2 |
|  | Student | 1 | 0 |
| **Characteristics of participants for the HCW-IDI (n=4)** | | | |
|  | (n=4) |  |  |
| Age | 25 to 31 |  |  |
| Religion | Islam (All) |  |  |
| Qualification | Diploma (All) |  |  |
| Year of experience | 2 to 5 years |  |  |
| Marital status | 1 Married, 3 Single |  |  |

**Supplementary Table 2: Caregiver’s recall by child’s age**

|  | **Has the caregiver confirmed that the vaccine card is accurate** | | |  |
| --- | --- | --- | --- | --- |
| **Child’s age** | Not accurate  N (row %) | Accurate  N (row %) | Not sure  N (row%) | Total  N (%) |
| 0-11 months | 65 (5.5) | 1068 (90.4) | 48 (4.1) | 1181 (100) |
| 12-23 months | 141 (13.7) | 844 (81.9) | 45 (4.4) | 1030 (100) |
| 24-59 months | 235 (11.5) | 1697 (82.9) | 115 (5.6) | 2047 (100) |
|  |  |  |  | Chi-square p-value= <0.001 |
